# Serum Proteomic Profiles of Patients with High and Low Risk of Endometrial Cancer Recurrence

**DOI:** 10.3390/ijms241914528

**Published:** 2023-09-25

**Authors:** Dagmara Pietkiewicz, Mikołaj Piotr Zaborowski, Kamila Jaz, Eliza Matuszewska, Agata Światły-Błaszkiewicz, Tomasz Kluz, Zenon J. Kokot, Ewa Nowak-Markwitz, Jan Matysiak

**Affiliations:** 1 Department of Inorganic and Analytical Chemistry, Poznan University of Medical Sciences, 3 Rokietnicka Street, 60-806 Poznan, Poland; eliza.matuszewska@ump.edu.pl (E.M.); jmatysiak@ump.edu.pl (J.M.); 2 Gynecologic Oncology Department, Poznan University of Medical Sciences, 33 Polna Street, 60-535 Poznan, Poland; mzaborowski@ump.edu.pl (M.P.Z.); kamila.jaz@wp.pl (K.J.); ewamarkwitz@ump.edu.pl (E.N.-M.); 3 Institute of Bioorganic Chemistry, Polish Academy of Sciences, Zygmunta Noskowskiego 12/14, 61-704 Poznan, Poland; 4 Department of Inorganic and Analytical Chemistry, Faculty of Pharmacy, Collegium Medicum in Bydgoszcz, Nicolaus Copernicus University in Toruń, Jurasza 2, 85-089 Bydgoszcz, Poland; agata.swiatly-blaszkiewicz@cm.umk.pl; 5 Department of Gynaecology, Gynaecologic Oncology and Obstetrics, Institute of Medical Sciences, Medical College of Rzeszow University, Rejtana 16c Street, 35-959 Rzeszow, Poland; jtkluz@interia.pl; 6 Faculty of Health Sciences, Calisia University, 13 Kaszubska Street, 62-800 Kalisz, Poland; z.kokot@akademiakaliska.edu.pl

**Keywords:** proteomics, endometrial cancer, mass spectrometry, high-risk endometrial cancer

## Abstract

Endometrial cancer is the most common gynecological cancer worldwide. Classifying endometrial cancer into low- or high-risk groups based on the following features is recommended: tumor grade, lymphovascular space invasion, myometrial involvement, and non-endometrioid histology. Despite the recent progress in molecular profiling of endometrial cancer, a substantial group of patients are misclassified based on the current criteria. This study aimed to identify proteins that could be used as biomarkers for the stratification of endometrial cancer patients into low- or high-risk groups. The proteomic analysis of serum samples from endometrial cancer patients was performed using matrix-assisted laser desorption/ionization–time of flight mass spectrometry (MALDI-TOF MS). The data were then analyzed using chemometric algorithms to identify potential biomarkers. Nineteen precursor ions were identified as fragments of eighteen proteins which included (1) connective tissue matrix proteins, (2) cytoskeletal proteins, and (3) innate immune system molecules and stress proteins. These biomarkers could be used to stratify the high- and low-risk patients, thus enabling more precise treatment decisions.

## 1. Introduction

Endometrial cancer (EC) is the most common gynecological cancer worldwide [1]. Although many patients with EC have a favorable prognosis, there is a significant subgroup with recurrent disease that poorly responds to treatment (high-risk group). Precisely identifying those patients and intensifying first-line therapy in this population is currently a clinical challenge. Tumors classified as high-risk diseases will require adjuvant treatment, including radiation therapy. Some of these patients will also benefit from chemotherapy. EC includes tumors of various histologies: endometrial (adenocarcinoma and its variants) and non-endometrioid cancers (mucinous, serous, clear cell, undifferentiated carcinoma, neuroendocrine tumors, and mixed carcinoma) [1]. The majority of patients (75–90%) with endometrial cancer have abnormal uterine bleeding at an early stage of disease [2,3]. The diagnosis of endometrial cancer in the absence of postmenopausal bleeding is not related to better survival compared to that of symptomatic patients [2]. For these reasons, the American Cancer Society does recommends any additional screening method for the early detection of endometrial cancer [4]. The screening strategy in the general population is to encourage all women at menopause to report any vaginal bleeding or discharge to a health professional [4]. In patients with Lynch syndrome, the screening is based on an annual endometrial biopsy starting at the age of 35 years [5]. Currently, no tests for the early detection of endometrial cancer are recommended in the general population, and there is no evidence that this approach changes patients’ prognosis. On the other hand, one of the main clinical challenges is identifying the patients at high risk of recurrence within the group of all patients with endometrial cancer. The management and prognosis significantly differ for these patients. This study aimed to investigate the potential of mass spectrometry in the stratification of endometrial cancer patients into low- and high-risk groups.

Although the histopathological type is related to prognosis, it has been revealed that other factors, such as pathological features and molecular profiling, correspond [6]. Tumors classified as high-risk diseases will require adjuvant treatment, including radiation therapy. Some of these patients will also benefit from chemotherapy.

The PORTEC-3 study defined a group of patients with high-risk disease (FIGO stage IA grade 3 with lymphovascular space invasion; stage IB grade 3 disease; stage II disease; stage IIIA, IIIB, and IIIC disease; or stage IA–III with serous or clear cell histology) who achieved an improvement in overall survival with chemoradiotherapy versus radiotherapy alone [1]. It has been established that patients with stage III of the disease or serous cancers may achieve the greatest benefit in overall survival [1]. At the same time, significantly more treatment-related long-term adverse events were reported in the chemoradiotherapy group, reflecting the need to identify factors that will allow for the selection of patients who can achieve the greatest benefit [1].

Better identification of the risk groups can be achieved by including the molecular profiling, following the 5th edition of the World Health Organization (WHO) Classification of Tumors of the Female Genital Tract, published in 2020 [7]. There are four molecular subtypes [8]. The DNA polymerase epsilon (*POLE*)-mutated subtype has a very high mutation frequency and elicits a robust immune response. Although its pathologic features are usually aggressive, it has highly favorable outcomes and is considered a low-risk group [9]. The mismatch repair-deficient subtype (MMRd) has a high mutational burden due to the dysfunction in mismatch repair proteins (MLH1, PMS2, MSH2, and MSH6). These tumors are susceptible to immune checkpoint inhibitors [10]. The P53 abnormal subtype has high copy number alterations due to mutations in the *TP53* gene. Regardless of histological features, these patients have a poor prognosis. The no specific molecular subtype is characterized by a low mutational burden, rare copy-number alterations, and functional mismatch repair mechanisms. These patients have intermediate to favorable outcomes [8].

The re-evaluation of samples from the PORTEC-3 trial enabled the exploration of the molecular differences in the group of patients classified as high-risk. The TransPORTEC consortium highlights the role of molecular analysis in stratifying patient risk [11]. It has been demonstrated that the benefit of incorporating adjuvant therapy can be predicted by considering the molecular alterations of the tumor, irrespective of risk category [12]. When applying the molecular analysis within the high-risk group, it is possible to distinguish the tumors with good (POLE-mutated tumors) or poor prognosis (p53 abnormal tumors) [5]. Therefore, it is proposed to combine molecular and histopathological features for patient stratification [5].

Despite the recent progress in molecular profiling of endometrial cancer, a substantial group of patients are misclassified based on the current criteria. Consequently, some of them receive inadequate treatment and risk having a relapse of malignancy. Conversely, others will suffer from the side effects of unnecessary therapy. It is highly important to stratify patients into low- and high-risk groups from a clinical point of view. First, the preoperative stratification may change the extent of surgery as resection of the lymph nodes for prognostic purposes is indicated in high-risk patients [5]. A systemic lymphadenectomy bears the risk of lymphedema and, therefore, is intended to be omitted if unnecessary [13]. Second, the high-risk patients will benefit from radiation and chemotherapy. These treatment modalities lead to significant long-term side effects that should be avoided in low-risk groups [1]. Third, a recent study has shown that patients with primary advanced endometrial cancer have survival benefits from immunotherapy [10]. Therefore, there is still a pressing need for better biomarkers to distinguish between low- and high-risk diseases to improve patients’ outcomes. 

Proteomics is a promising approach to identifying proteins that can be used as new targets for therapeutic interventions and as markers for early cancer detection. In this study, we intended to test whether patients classified as having a high-risk disease (according to the PORTEC3 study) have a serum proteomic profile that is distinctive from that of the low-risk group. In the future, such a pattern could be sought within the presumptive low-risk group to identify patients who are at a higher risk of recurrence. We demonstrate that the mass spectrometry analysis of the serum from peripheral blood can differentiate the above-mentioned patient subgroups. Therefore, this method may help better identify low- and high-risk patients with endometrial cancer. In addition, the detected proteins could also be candidate drug discovery targets.

To the best of our knowledge, this is the first study utilizing MALDI-TOF MS (matrix-assisted laser desorption/ionization–time of flight mass spectrometry) combined with solid-phase extraction pretreatment for the proteomic identification of potential biomarkers that can predict high-risk endometrial carcinoma. The MS data were analyzed with advanced chemometric tools, and a classification model with the highest recognition capability for patients with high and low risk of endometrial cancer recurrence was calculated. The identification of potentially differentiating proteins was performed using nano-LC (nano-liquid chromatography) coupled with MALDI-TOF-MS/MS.

## 2. Results

Patients were divided into low- and high-risk groups following the criteria from the PORTEC-3 study. The high-risk group included FIGO stage IA grade 3 with lymphovascular space invasion; stage IB grade 3 disease; stage II disease; stage IIIA, IIIB, and IIIC disease; or stage IA–III with serous or clear cell histology. The other patients were considered a low-risk group. In this study we included 115 patients; 52 patients were classified as patients with a high risk of endometrial cancer recurrence, and 63 patients were classified as patients with a low risk of endometrial cancer recurrence. The epidemiological and clinical data characterizing the low- and high-risk groups are presented in Table 1.

The obtained spectra were statistically analyzed in the chemometric program ClinProTools 3.0 (Bruker Daltonics, Bremen, Germany). Since each sample was analyzed by mass spectrometry three times, a grouping function was used to obtain one average repetition for a given sample. A comparison of the obtained data was achieved through a standard workflow. Each spectrum was first normalized to the total ion current (TIC) and recalibrated; the “top hat” baseline subtraction with the minimum baseline width set to 10% was used to remove broad structures. Spectra were also smoothed and processed in the mass range of 1000–10,000 Da with the selection of peaks with a signal-to-noise ratio above 5 and their intensity was calculated. Spectra were analyzed in the mass range from 1 to 10 kDa. The total mean spectra for the individual groups were calculated from the pre-prepared spectra.

**Figure 1:**
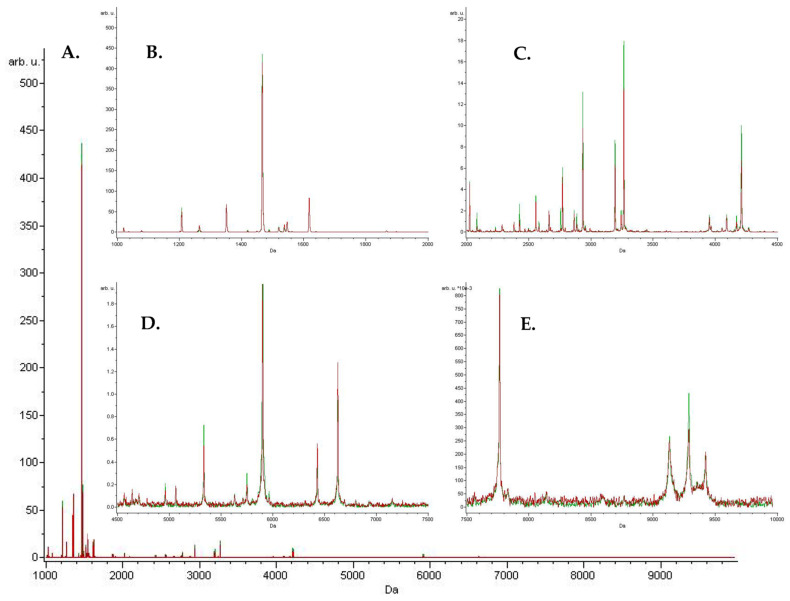
Total average MALDI-TOF MS spectra of serum samples derived from study participants over the full scan range of *m*/*z* 1–10 kDa (**A**); zoomed over the *m*/*z* 1–2 kDa range (**B**); zoomed over the *m*/*z* 2–4.5 kDa range (**C**); zoomed over the *m*/*z* 4.5–7.5 kDa range (**D**); and zoomed over the *m*/*z* 7.5–10 kDa range (**E**); red—high risk; green—low risk.

The total average MALDI-TOF MS spectra of the studied groups are shown in Figure 1. The use of chemometric algorithms offered by ClinProTools led to the selection of precursor ions to differentiate the study groups. The peaks that could discriminate the study groups that were generated by the applied algorithms are shown in Table 2. Some peaks are present in both the genetic algorithm (GA) and quick classifier (QC) algorithms. They were featured with an *m*/*z* of 1073.18, 6432.36, 1202.48, 1520.06, and 1993.93. The peaks of *m*/*z* 1537.99 and 3241.62 were classified as a differentiator in both the GA- and Supervised Neural Network (SNN)-based models. The m/z peaks of 3192.65, 4209.97, and 6630.42 were selected in both the QC and SNN models.

The highest value of average cross-validation (83.07%) and the recognition capability (96.93%) from the three replicates were obtained using the genetic algorithm (Table 3). The separate average spectra for each group are available as Supplementary Materials (Figures S1 and S2).

The highest value of average cross-validation (83.07%) and the recognition capability (96.93%) from the three replicates were obtained using the genetic algorithm (Table 3).

### Peaks Identification

The methodology used in this study allowed us to identify nineteen precursor ions as fragments of eighteen proteins. The identification of intact proteins was based on their peptide fragments with a mass not exceeding 10 kDa. This was due to the better resolution of the MALDI-TOF/TOF mass spectrometer in the lower mass range. A list of identified ions, together with their measured masses, protein fragmentation amino acid sequence, IDs, and names of proteins as fragments from which the selected ions have been identified, is presented in Table 4. The MALDI-TOF/TOF MS identification was conducted in the mass range of m/z 700–3500. Therefore, the peaks exceeding this range could not be identified. Also, some peaks within the mass range were not identified, likely because of insufficient resolution and the presence of neighboring peaks. These ions require further experiments. The upregulated and downregulated proteins were determined based on relative peak intensities. 

## 3. Discussion

Our research reveals differences in serum protein composition between high- and low-risk endometrial cancers. In our study, we identified 19 discriminatory proteins [14,15,16]. The fragments of proteins that we identified can be divided into three main groups: (1) connective tissue matrix proteins (collagen alpha-1(I) chain, fibrinogen gamma chain, and inter-alpha-trypsin inhibitor heavy chain H2) [17], (2) cytoskeletal proteins (myosin-9, tropomyosin beta chain, actin-related protein 3, alpha-actinin-4, actin, cytoplasmic 1, transgelin-2, and vimentin), and (3) innate immune system molecules and stress proteins (complement C3, heat shock protein HSP 90-alpha, 78 kDa glucose-regulated protein, and heat shock 60 kDa protein 1) [17].

Some of the proteins we detected are components of the extracellular matrix (ECM) [12,15]. We identified the collagen alpha-1(I) chain as upregulated in serum in the high-risk group. We hypothesize that there are two possible mechanisms of higher levels of collagen in the serum of the high-risk group. First, it is possible that it accumulates in response to the stimulation by the cancer-associated fibroblasts, which, consequently, creates a stiffening of the ECM and increases the cytoskeletal tension. The dysregulation of the ECM composition alters the cytoskeletal structure and promotes tumor metastasis [16]. Recently, we observed that components of ECM stimulate invasiveness and proliferation in ovarian cancer models [18]. Second, it is possible that ECM proteins, such as collagen, are released into the peripheral blood as a result of tissue digestion by metalloproteinases that are secreted by more invasive cancer cells. It has been demonstrated that fibrinogen is expressed in endometrial cancer [19]. Higher expression of fibrinogen was also identified in the high-risk patients in our study. As a part of the tumor stroma, fibrinogen may promote tumor proliferation and, by binding to the vascular growth factors, may stimulate tumor neovascularization in endometrial cancer [19]. Inter-alpha-trypsin inhibitor heavy chain H2 contributes to ECM stability [20]. Consistent with our findings, it was reported to be downregulated in many solid tumors, including endometrial cancer [20].

Interestingly, many (7 out of 19) of the discriminatory proteins in our study belong to cytoskeleton components. We showed that multiple components of microfilaments are deregulated. The cytoplasmic actin, alpha-actinin-4, and myosin-9 were more prevalent in the serum of the low-risk group [16]. This aligns with a previous study which showed that alpha-actinin 4 was lower in endometrial cancers with lymph node metastases that are considered high-risk diseases [21]. Actin plays an important protective role as a regulator of oxidative stress by influencing intracellular reactive oxygen species levels. Previous studies have demonstrated that oxidative stress induces the response via the p53 protein, which inhibits the contractibility of the actin-related protein actomyosin via Rho kinases [16]. Another actin-binding cytoskeletal protein that we identified is tropomyosin. Tropomyosin is well known as a part of the muscle cell contraction complex, and its function in actin filament organization contributes to stabilizing the microfilament bundles [22,23]. A decrease in tropomyosin synthesis has been observed in cells exposed to factors that trigger DNA damage [23]. The tropomyosin inhibitors, for example, tropomyosin inhibitor ATM-3507, prolonged vinorelbine-induced mitotic arrest in ovarian cancer cells [24]. We demonstrated that another actin-binding protein, transgelin-2, can be used to discriminate between high- and low-risk patients. Transgelin-2 plays a role in stabilizing the immune cell structure during its interaction with antigen-presenting cells as a part of the cytoskeletal structure of T and B lymphocytes. Both up- and downregulation of transgelin-2 may promote metastasis in various types of cancers [25]. The deregulation of actin and its binding proteins implies that intense cytoskeleton remodeling is a discriminatory feature between low- and high-risk endometrial cancer. Some fragments of the cellular cytoskeleton may also appear in the peripheral blood due to the digestion of the smooth muscles of the uterus as endometrial cancer invades the uterine wall.

We detected peptides representative of vimentin, a component of a different class of cytoskeleton proteins that form intermediate filaments. We observed that in the high-risk group, vimentin levels tended to be lower in the serum. Vimentin is important for cell motility [26]. In many cancer types, an increase in vimentin and a decrease in E-cadherin were reported during epithelial-to-mesenchymal transition (EMT) [27]. The EMT process enables the cancer cells to acquire a mesenchymal phenotype, which leads to higher invasiveness and facilitates metastasis [28]. However, such a relationship was not observed in endometrial cancer [29]. Surprisingly, low epithelial vimentin expression was reported in endometrial cancer with a high risk of recurrence, as confirmed in 518 samples by immunohistochemistry [29]. In accordance with this study, we detected lower levels of vimentin in high-risk patients. Our observation might be clinically applicable, as vimentin measured non-invasively in serum could stratify patients prior to surgery.

We found that a group of innate immune system molecules and stress proteins were also discriminatory. Heat shock proteins (HSPs) comprise several families whose role lies in the induction of immune response caused by cellular stress. Various types of immune cells, including natural killer (NK) cells, T cells, monocyte-derived dendritic cells (mDCs), and neutrophils, produce HSPs. These proteins trigger anti-tumor responses and modulate the activation of immune cells [30]. The HSP complex can induce the anti-tumor response by activating cytotoxic lymphocytes [14]. Primary advanced or recurrent endometrial cancer patients can be treated with immunotherapy [10]. It is possible that tracking HSPs in the serum could help identify responders to this type of therapy. We also found a complement-C3 protein fragment, a part of the complement system cascade engaged in an innate immune response. The complement system activation leads to the formation of a membrane attack complex, which is responsible for antibody-mediated cytotoxicity. C3 secretion has been shown to promote tumor progression and metastases by regulating the function of ECM proteins [31]. 

Proteome-wide profiling remains to be implemented as a powerful tool in up-to-date science. Our previous studies confirmed that this approach is accurate for the characterization and identification of proteomic patterns of different diseases. Identifying high-risk endometrial patients is an important clinical problem. It is possible that mass spectrometry will enhance patient stratification. However, it is not free of limitations. The above-mentioned findings warrant further experimentation and clinical validation in larger cohorts of EC patients. Moreover, a quantitative approach should be applied in further studies to confirm the results of the peptide–protein profiling. Also, further studies are planned to identify the remaining discriminative peaks since they might extend our knowledge of EC recurrence.

## 4. Materials and Methods

### 4.1. Study Groups and Sample Collection

All patients provided a signed informed consent, which was approved by the Ethics Review Board of Medical Chamber in Rzeszów (Consent No 90/B/2016). The study included sera collected from 115 patients with malignant uterine cancer treated at the Clinical Gynecology and Obstetrics Department of the Wojewódzki Szpital nr 1 im. Fryderyk Chopin in Rzeszów in 2016–2017. The mean age of the patients was 64 years. Blood was collected from patients diagnosed with endometrial cancer based on endometrial biopsy. The samples were collected before surgery. The criterion for inclusion in the study was a diagnosis of endometrial carcinoma of the endometrium (EC). Patients were divided into low- and high-risk groups following criteria from the PORTEC-3 study. The high-risk group included FIGO stage IA grade 3 with lymphovascular space invasion; stage IB grade 3 disease; stage II disease; stage IIIA, IIIB, and IIIC disease; or stage IA–III with serous or clear cell histology. The other patients were considered the low-risk group. A total of 52 patients were classified as patients with a high risk of endometrial cancer recurrence, and 63 patients were classified as patients with a low risk of endometrial cancer recurrence.

### 4.2. Serum Samples Pretreatment

Prior to performing the MALDI-TOF MS analyses, the samples were prepared using ZipTip C18 tips according to the manufacturer’s protocol (Millipore, Bedford, MA, USA) to desalt and concentrate the samples. ZipTip C18 tips use a solid-phase extraction (SPE) technique. In this technique, proteins and peptides bind to the C18 bed, but salts, lipids, and other contaminants, as well as excess high-abundance proteins, are removed. This approach reduces the masking of low-abundance proteins by high-abundance proteins and allows for more comprehensive proteomic analysis results. The serum samples were diluted in 0.1% trifluoroacetic acid (TFA) in water (1:5 ratio) to obtain a 10 uL sample suitable for the ZipTip procedure. ZipTip pipette tips were conditioned with acetonitrile (ACN) and 0.1% TFA. Peptides were bound to the equilibrated ZipTip pipette tips by performing aspirate–dispense cycles of the entire sample. After washing in 0.1% TFA, the bound peptides were eluted with 4 μL of 50% ACN in 0.1% TFA.

### 4.3. MALDI-TOF Proteomic Profiling

After ZipTip purification and initial concentration of samples, MALDI-TOF MS analysis was performed as previously described [32]. A 1 μL volume of each eluent sample was mixed with 10 μL of a 0.3 g/L α-cyano-4-hydroxycinnamic acid (HCCA) matrix solution in ethanol/acetone (2:1, *v*/*v* ratio). The mixture was applied onto an AnchorChip Standard 800 μm target plate (Bruker Daltonics, Bremen, Germany) in triplicate and allowed to crystallize at room temperature. To perform MS analyses, a MALDI-TOF/TOF UltrafleXtreme mass spectrometer (Bruker Daltonics, Bremen, Germany) was used. The measurements were performed in the positive linear mode; 2000 laser shots were acquired per sample in the *m*/*z* 1000–10,000 range. MS spectra were externally calibrated with a mixture of a protein I calibration standard and a peptide calibration standard (BrukerDaltonics, Bremen, Germany) (5:1, *v*/*v*). The average mass deviation from the reference masses was less than 100 ppm. To suppress unwanted ions, the matrix suppression parameter was set at 700 Da. The MS parameters for analysis were as follows: pulsed ion extraction, 260 ns; lens, 6.40 kV. FlexControl 3.4 software (Bruker Daltonics, Bremen, Germany) was used for the acquisition and processing of MS spectra.

### 4.4. Data Analysis

The obtained spectra were statistically analyzed in the chemometric program ClinProTools 3.0 (Bruker Daltonics). Since each sample was analyzed by mass spectrometry three times, a grouping function was used to obtain one average repetition for a given sample.

Three mathematical algorithms were used to create discriminant models: GA, SNN, and QC. The algorithms allowed us to obtain groups of peaks with the greatest discriminating ability. Two parameters were calculated for the models: recognition capability and cross-validation. The results obtained in the present analysis were cross-validated using the “leave one out” method. In this method, one of the results obtained is excluded (the others form the “learning” set) and was then used as the “test” set. The size of the study and control groups dictated the choice of this method. The recognition capability expresses the percentage of subjects correctly assigned to the test and control groups. 

Each model showed a combination of differentiating peaks. A detailed description of all chemometric algorithms is included in our previous publication [33].

### 4.5. NanoLC-MALDI-TOF/TOF MS Discriminative Peaks Identification

The detailed protocol used for identifying the discriminative peaks using a nanoLC- MALDI-TOF/TOF MS (Bruker Daltonics, Bremen, Germany) platform has been described in our previous paper [33]. For the identification analysis, we pooled the same samples used for protein–peptide profiling. In brief, the serum samples subjected to analyses were first purified using ZipTip C18 micropipette tips. The obtained eluates in a volume of 4 µL were subjected to nanoLC separation. The obtained fractions were mixed with HCCA matrix solution and automatically spotted onto an AnchorChip 384 MALDI target plate (Bruker Daltonics, Bremen, Germany). The MS and MS/MS experiments were performed in the reflectron positive ion mode in the mass range of *m*/*z* 700–3500. Proteomic identification was based on the MS/MS fragmentation spectra, the SwissProt database, and the Mascot 2.4.1 search engine. The database searches were taxonomically restricted to Homo sapiens.

## 5. Conclusions

This study utilizing MALDI-TOF MS combined with solid-phase extraction pretreatment to analyze plasma samples from patients with EC highlighted the potential of proteomic analysis to identify biomarkers for risk stratification in endometrial cancer and improve treatment selection. While nineteen precursor ions representing eighteen proteins were identified as potential biomarkers, their utility as biomarkers of high-risk EC requires further investigation.

## Figures and Tables

**Table 1:** Epidemiological and clinical characteristics characterizing low- and high-risk groups. Values represent medians and interquartile ranges. BMI—body mass index, FIGO—International Federation of Gynecology and Obstetrics (FIGO) staging system.

Group	Low Risk	High Risk
Number of patients	67	52
Age [years]	64 (60–70.5)	65 (61–72)
BMI [kg/m^2^]	32.1 (28.2–37.2)	28.9 (26.7–33.1)
FIGO stage		
IA	49	6
IB	18	7
II	0	20
III	0	16
IV	0	3
Grade		
1	41	9
2	23	20
3	3	22
Histology		
Endometroid	67	33
Non-endometroid	0	19

**Table 2:** Peaks discriminating study groups generated by the applied algorithms.

GA	QC	SNN
2661.15 2284.03 1073.18 6432.36 3278.62 1568.47 3241.62 1866.53 1202.48 1520.06 1373.53 2294.99 1537.99 4091.16 1993.93	1036.97 1073.18 1202.48 1520.06 1993.93 3192.65 3263.71 4209.97 5904.21 6432.36 6630.42	1207.39 1528.12 2080.94 1537.99 2933.38 3241.62 1546.67 3192.65 2884.59 1639.7 4209.97 2579.98 1419.82 4170.79 1510.74 3215.33 7765.27 6630.42 4149.28 1264.4

**Table 3:** Values of chemometric parameters for the algorithms of the comparison of patients with high and low risk of endometrial cancer recurrence.

	GA	QC	SNN
Cross-validation (%)	**83.07**	58.11	67.58
Recognition capability (%)	**96.93**	63.72	56.87

GA—genetic algorithm, QC—quick classifier, SNN—supervised neural network.

**Table 4:** List of precursor ions identified as fragments of proteins. ↑—up-regulated protein; ↓—down-regulated protein.

Precursor Ion *m/z*	Protein Fragmentation Sequence	UniProtKB-ID	Protein Name	Protein Expression in High-Risk Patients Compared to Low-Risk Patients
2661.15	K.ANQQFLVYCEIDGSGNGWTVFQK.R	FIBG_HUMAN	Fibrinogen gamma chain	↑
2284.03	R.EIEDPEDRKPEDWDERPK.I	CALX_HUMAN	Calnexin	↑
1073.18	K.LDKENAIDR.A	TPM2_HUMAN	Tropomyosin beta chain	↑
1568.47	H.GHEQQHGLGHGHKF.K	KNG1_HUMAN	Kininogen-1	↑
1866.53	M.AGRLPACVVDCGTGYTK.L	ARP3_HUMAN	Actin-related protein 3	↑
1202.48	K.NVIGLQMGTNR.G	TAGL2_HUMAN	Transgelin-2	↑
1520.06	R.KTFTAWCNSHLR.K	ACTN4_HUMAN	Alpha-actinin-4	↓
2294.99	R.IQEIIEQLDVTTSEYEKEK.L	CH60_HUMAN	60 kDa heat shock protein, mitochondrial	↑
1537.99	R.FAIQDISVEETSAK.E	ACTN1_HUMAN	Alpha-actinin-1	↑
1993.93	K.TGPPGPAGQDGRPGPPGPPGAR.G	CO1A1_HUMAN	Collagen alpha-1(I) chain	↑
1036.97	K.RLDGSVDFK.K	FIBG_HUMAN	Fibrinogen gamma chain	↑
1528.12	R.AKFEELNMDLFR.S	GRP78_HUMAN	78 kDa glucose-regulated protein	↓
2080.94	K.SMEAEMIQLQEELAAAER.A	MYH9_HUMAN	Myosin-9	↓
1546.67	K.LKECCEKPLLEK.S	ALBU_HUMAN	Serum albumin	↑
1639.7	R.LDLAGRDLTDYLMK.I	ACTB_HUMAN	Actin, cytoplasmic 1	↓
2579.98	K.QKPDGVFQEDAPVIHQEMIGGLR.N	CO3_HUMAN	Complement C3	↓
1419.82	K.VQFELHYQEVK.W	ITIH2_HUMAN	Inter-alpha-trypsin inhibitor heavy chain H2	↓
1510.74	R.MFGGPGTASRPSSSR.S	VIME_HUMAN	Vimentin	↓
1264.4	R.RAPFDLFENR.K	HS90A_HUMAN	Heat shock protein HSP 90-alpha	↑

## Data Availability

The data presented in this study are available on request from the corresponding author.

## Supplementary Materials

The supporting information can be downloaded at: https://www.mdpi.com/article/10.3390/ijms241914528/s1.
