# Supplementary material for: Serum Proteomic Profiles of Patients with High and Low Risk of Endometrial Cancer Recurrence
Source: Int J Mol Sci. 2023 Sep 25;24(19):14528. doi: 10.3390/ijms241914528 (PMC10572223; doi:10.3390/ijms241914528)
Supplement: Supplementary file 1 [file ijms-24-14528-s001.zip › ijms-2564590-supplementary.pdf]

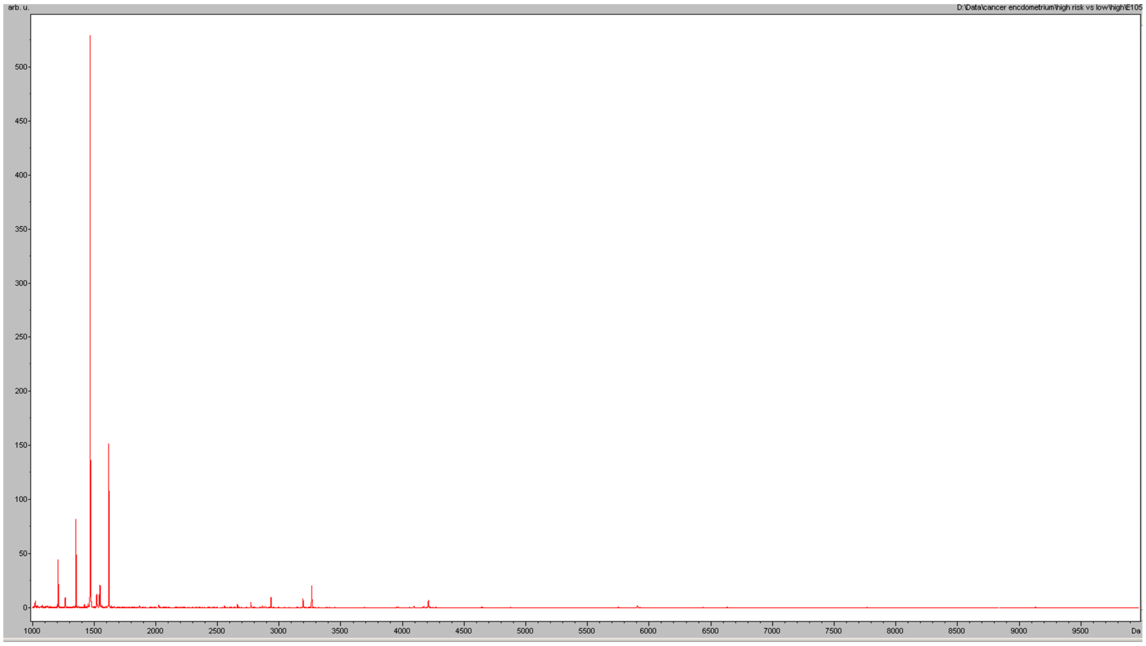

Figure S1. Averaged spectra for high-risk endometrial cancer recurrence group in the mass range 1000-10,000 Da.

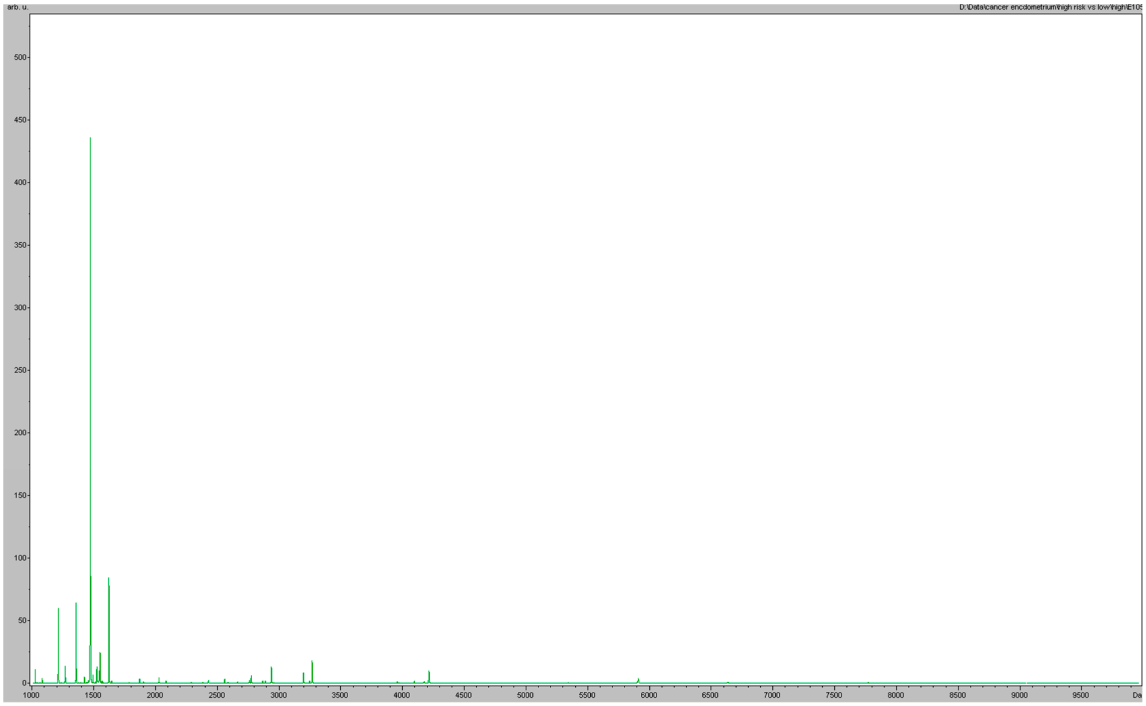

Figure S2. Averaged spectra for low-risk endometrial cancer recurrence group in the mass range 1000-10,000 Da.
